# Natural Cytotoxicity Receptors in Decidua Natural Killer Cells of Term Normal Pregnancy

**DOI:** 10.1155/2018/4382084

**Published:** 2018-08-01

**Authors:** Hideki Takahashi, Tatsuo Yamamoto, Motomi Yamazaki, Takayuki Murase, Takayuki Matsuno, Fumihisa Chishima

**Affiliations:** Department of Obstetrics & Gynecology, School of Medicine, Nihon University, Tokyo, Japan

## Abstract

**Aim:**

To investigate the changes in the maternal immune system at term pregnancy, we studied the expression of natural cytotoxicity receptors (NCRs) and the cytokine production of NK cells in term placenta decidua and peripheral blood.

**Methods:**

Term decidua and peripheral blood were taken from patients undergoing elective cesarean section. The lymphocytes were separated using density gradient centrifugation (DGC) from peripheral blood and were separated from decidua using DGC after enzyme digestion. These cells were stained with FITC anti-CD56 and Per-CP anti-CD3 monoclonal antibodies, and the NCRs were stained with PE-conjugated anti-NKG2D, NKp46, NKp30, and NKp44 monoclonal antibodies. Cytokines, including IFN-*γ*, TNF-*α*, IL-10, and TGF-*β*, were stained and then analyzed by flow cytometry.

**Results:**

There were fewer cells positive for NKG2D, NKp46, and NKp30 among CD56+CD3- cells in deciduas than in peripheral blood, but the percentages of NKp44-positive cells in CD56+CD3- lymphocytes in deciduas tended to be higher.

**Conclusion:**

The decreased expression of some NCRs in deciduas may be related to decreased cytotoxicity at term pregnancy, but the increased expression of NKp44 may affect the increased cytokine production in the decidua. Similarly, the expression of NCRs in the decidua may be connected to the maintenance of pregnancy at term.

## 1. Introduction

In successful pregnancy, there are many mechanisms by which the fetus avoids rejection by the maternal immune system [1]. It has been reported that the cytotoxic activity of natural killer (NK) cell is depressed during pregnancy and that the suppression of cytotoxicity is multifactorial and may include both cellular and humoral elements [2]. Uterine decidual cells have depressed NK cytotoxicity [3–5]. It has been reported that the killer inhibitory receptors (KIR) in uterine NK cells play a role in maintaining a normal pregnancy. HLA-G, as one of the HLA class I antigens, is expressed in extravillous trophoblasts, and NK cytotoxicity is suppressed by stimulation of KIR by HLA-G [6]. However, the elements of suppressed cytotoxicity have not been evaluated well.

The receptors that are responsible for NK cell activation during the process of natural cytotoxicity are collectively termed natural cytotoxicity receptors (NCRs). NCRs consist of CD335 (NKp46), CD336 (NKp44), CD337 (NKp30), and CD314 (NKG2D) [7]. NKp46 and NKp30 are expressed either in resting or in activating NK cells, while NKp44 is expressed only in activated NK cells. The ligands recognized by NCRs are still incompletely molecularly defined. NKp46 and NKp44 detect the terminal N-acetylneuraminic acid residues (sialic acids) in sendai virus neuraminidase and influenza virus haemagglutinin. NKp44 detects cell-free mycobacteria and HIVgp41 [8].

Marlin R reported on NCR manifestations, such as NKp46, NKp30, NKp44, and NKG2D of CD56+CD3- NK cells in the decidua during the first trimester of pregnancy [9].

However, the NCRs that appear at term pregnancy have not been studied well.

Moreover, the role of cytokines in the decidua is important in the maintenance of pregnancy [10–13]. The relationships between NCRs and the intracellular cytokine expression of CD56+ NK cells in peripheral blood NK cells and the uterine endometrium have been analyzed [14, 15].

There are two kinds of NK cells: mature NK cells (CD56dim+CD16+, CD56+CD16+CD3-) and immature NK cells (CD56bright+CD16-, CD56+CD16-CD3-) [16]. Mature NK cells, which are present in peripheral blood, make up almost all NK cells, and, in the decidua, there is a large amount of immature NK cells that are known as uterine NK cells [17–19]. The cytokine production of CD56+CD3-CD16- NK cells is higher than that of CD56+CD3-CD16+ NK cells, which possibly gives the former a role in maintaining pregnancy [18, 20].

To investigate the changes in the immune system in term normal pregnancy, we first analyzed the subtypes of NK cells in peripheral blood and decidua during normal pregnancy; then, we analyzed the expression of NCRs on the CD56+CD3- NK cell; and, finally, we evaluated cytokine production in the peripheral blood and decidua in term pregnancy.

## 2. Material and Methods

After obtaining informed consent, maternal peripheral blood samples and decidua were taken from 19 cases of term normal pregnancy with elective cesarean section of normotensive pregnancies without complications. The protocol for the research project was approved by a suitably constituted Ethics Committee of the institution within which the work was undertaken, and it conformed to the provisions of the Declaration of Helsinki. After placenta removal, decidua samples were carefully swabbed from the uterine wall with gauze. The patients were not in labor, and there was no premature rupture of membranes. The average maternal age was 35.5 years (range: 20-44 years), and the average gestational period was 37.8 weeks (range: 37-39 weeks). The samples were measured within 6 hours.

### 2.1. Isolation of Decidua and Peripheral Blood Lymphocytes

Decidua cell suspensions were prepared from decidua tissues by a modification of Petrovic's method [21]. After the fragments of decidua were washed with PBS and the maternal blood was removed, the tissues were identified macroscopically and minced with scissors and then incubated at 37°C in 5% CO_2_ with continuous agitation in RPMI-1640 medium (GIBCO, GrandIsland, NY, USA) containing 0.25% Trypsin-EDTA (GIBCO). After 90 minutes, the cell suspension was filtered through a 100 *μ*m nylon mesh Corning Cell Strainer (CORNING, Tewksbury, MA, USA), washed three times with RPMI-1640, and centrifuged at 400 g. The cell pellets were resuspended in RPMI-1640 containing 10% fetal bovine serum (FBS) (SIGMA, St Louis, MO, USA) and then layered onto Lymphoprep (AXIS-SHILD, Oslo, Norway) solution and centrifuged for 20 minutes at 400 g. Cells aspirated from the interface were washed three times with PBS and adjusted to a concentration of 1X10^7^ cells/ml in RPMI. Cell viability was confirmed to be >95% by trypan blue dying. Peripheral blood lymphocytes were also separated using Lymphoprep.

### 2.2. Antibody Labeling

One hundred microliters of peripheral blood and decidua lymphocytes (1X10^6^ cells/100 *μ*l) was labeled with 20 *μ*l of FITC-conjugated anti-CD56 monoclonal antibody (Clone B159, Becton-Dickinson, Franklin Lakes, NJ, USA), PE-conjugated anti-CD314 (NKG2D) (Clone1D11, eBioscience, San Diego, CA, USA), anti-CD335 (NKp46) (Clone BAB281, BECKMAN COULTER, Brea, CA, USA), anti-CD336 (NKp44) (Clone Z231, BECKMAN), anti-CD337 (NKp30) (Clone Z25, BECKMAN), PerCP-conjugated anti-CD3 antibody (Clone SK7, Becton-Dickinson), and PerCP-conjugated anti-CD16 (Clone 3G8, BioLegend, San Diego, CA, USA).

### 2.3. Flow Cytometry

Antibody-labeled cells were analyzed by flow cytometry using a Fluorescence-Activated Cell Sorter (FACS) Calibur (Becton-Dickinson, Franklin Lakes, NJ, USA). The flow cytometer utilized an argon laser with 15 mW at 488 nm excitation. Gates were set on forward scatter (FSC) and side scatter (SSC) dot plots to exclude noncellular debris and focus on the lymphocytes. With the gate set around the lymphocytes, crossovers of fluorescent label 1 (FL1), FL2, and FL3 signals were adjusted by compensation using FITC-, PE-, or PerCP-conjugated antibodies. The results with antibodies were expressed as percentages of cells or lymphocytes. For each experiment, 1X10^5^ cells and lymphocytes were evaluated. The percentages of cells labeled antibody-positive were quantitated using CELLQuest (Becton-Dickinson), and the data were analyzed using the FlowJo software (Tree Star, Ashland, OR, USA).

### 2.4. Cytokine Detection

Flow cytometry was used to evaluate intracellular cytokines. The evaluation of the production of cytokine was made after the activation of PMA/ionomycin according to the method of Fukui et al. [14]. Isolated cells were incubated with phorbol 12-myristate 13-acetate (PMA) (25 ng/ml), ionomycin (1*μ*M) and Brefeldin-A (5 *μ*g/ml) for 4 hours. Activated cells were stained with conjugated anti-CD56 and anti-CD3 antibody and were permeabilized. Then, they were again stained with PerCP-conjugated anticytokine monoclonal antibodies specific to IFN-*γ* (Clone 4S.B3, BioLegend), TNF-*α* (Clone Mab11, BioLegend), IL-10 (Clone JES3-9D7, BioLegend), and LAP (TGF-*β*1) (Clone TW4-2F8, BioLegend).

### 2.5. Statistical Analyses

All values are expressed as mean±standard deviation (SD). The data were analyzed using* t*-tests or Mann–Whitney* U* tests with GraphPad Prism 6 (San Diego, CA, USA), with the level of confidence considered significant at P<0.05.

## 3. Results

**Figure 1:**
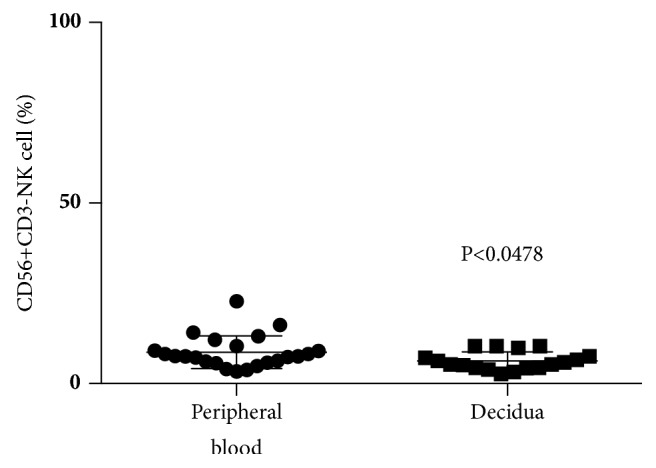
Percentage of CD56+CD3- cells among lymphocytes at term pregnancy in peripheral blood and decidua. The percentages of CD56+CD3- lymphocytes in decidual cells were lower than those in peripheral blood.

**Figure 2:**
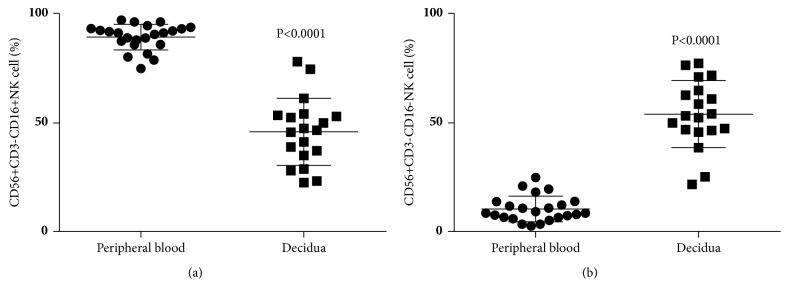
(a) Percentage of CD56+CD3-CD16+ cells among CD56+CD3- cells at term pregnancy in peripheral blood and decidua. The percentages of CD56+CD3-CD16+ lymphocytes in decidual cells were lower than those in peripheral blood. (b) Percentage of CD56+CD3-CD16- cells among CD56+CD3- cells at term pregnancy in peripheral blood and decidua. The percentages of CD56+CD3-CD16- lymphocytes in decidual cells were higher than those in peripheral blood.

We analyzed the distribution of NK cell subpopulations in peripheral blood and decidua. An analysis of CD56+CD3- cells (total NK cells) in CD56-positive lymphocytes at normal term pregnancy showed that the percentages of CD56+CD3- cells among CD56-positive lymphocytes in decidua cells (6.3±2.5%) were lower than those in peripheral blood (8.7±4.5%) (Figure 1). When CD56+CD3-CD16+ cells (mature NK cells) among CD56+ lymphocytes were analyzed under the same conditions, the percentages of CD56+CD3-CD16+ cells in CD56+ lymphocytes in the decidua (45.9±15.5%) were lower than those in peripheral blood (89.6±5.9%) (Figure 2(a)). When CD56+CD3-CD16- cells (immature NK cells) among CD56+ lymphocytes were analyzed under similar conditions, the percentages of CD56+CD3-CD16- cells among CD56+ lymphocytes in the decidua (54.1±15.5%) were higher than those in peripheral blood (10.4±5.9%) (Figure 2(b)).

**Figure 3:**
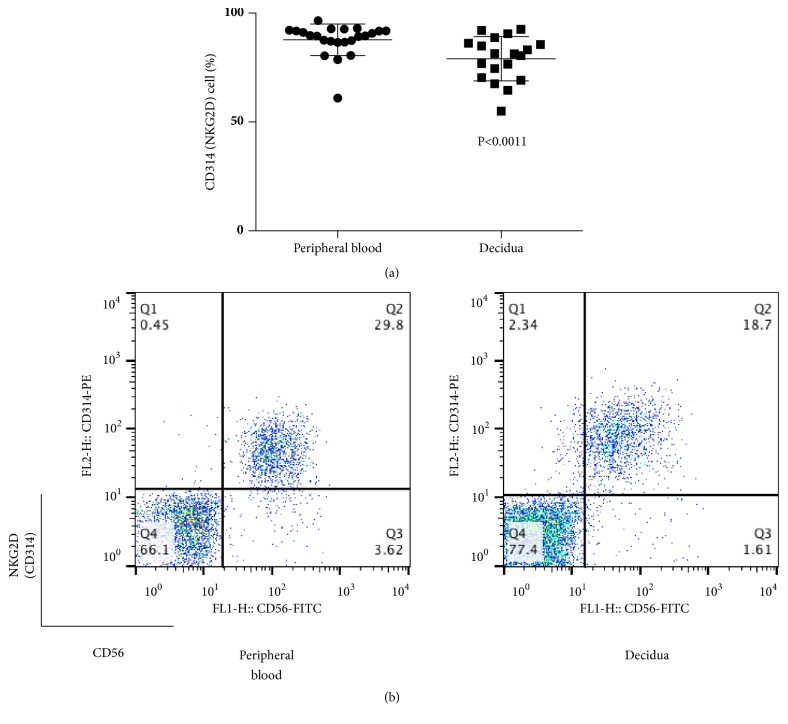
(a) Percentage of CD314 (NKG2D)+ cells among CD56+CD3− cells at term pregnancy in peripheral blood and decidua. The percentages of CD314 (NKG2D)+ lymphocytes in decidua were lower than those in peripheral blood. (b) Representative dot plot analysis of CD314 (NKG2D)+ cells among CD56+3- lymphocytes taken from term pregnancy by flow cytometry. The gate was set around the CD3- lymphocytes to analyze the CD314+CD56+ NK cells.

**Figure 4:**
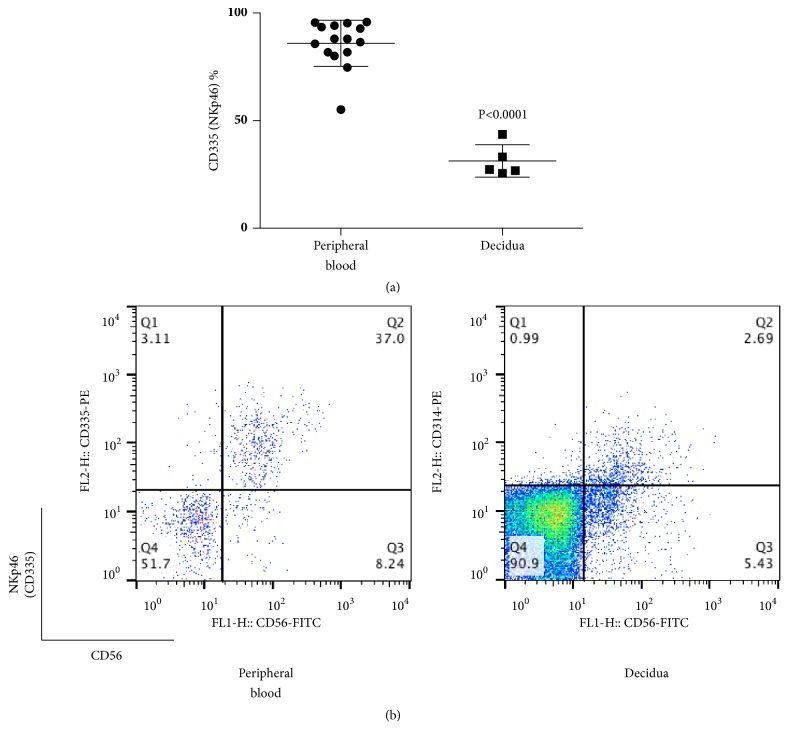
(a) Percentage of CD335 (NKp46)+ cells among CD56+CD3− cells at term pregnancy in peripheral blood and decidua. The percentage of CD335 (NKp46)+ lymphocytes in decidua was lower than in peripheral blood. (b) Representative dot plot analysis of CD335 (NKp46)+ cells among CD56+CD3- lymphocytes taken from term pregnancy by flow cytometry. The gate was set around the CD3- lymphocytes to analyze the CD335+CD56+ NK cells.

Next, we analyzed the percentages of NCRs of CD56+CD3- lymphocytes (total NK cells) in peripheral blood and decidua during normal pregnancy. The percentages of NKG2D (CD314)+ cells among CD56+CD3- lymphocytes were 79.0±10.2 and 87.7±7.3 in decidua and peripheral blood, respectively (Figure 3), while the percentages of NKp46 (CD335)+ cells among CD56+CD3- lymphocytes were 31.3±7.5 and 85.9±10.7 in decidua and peripheral blood, respectively (Figure 4). In both cases, the values in the decidua appear to be lower than those in peripheral blood (p≦0.0011, p≦ 0.0001).

**Figure 5:**
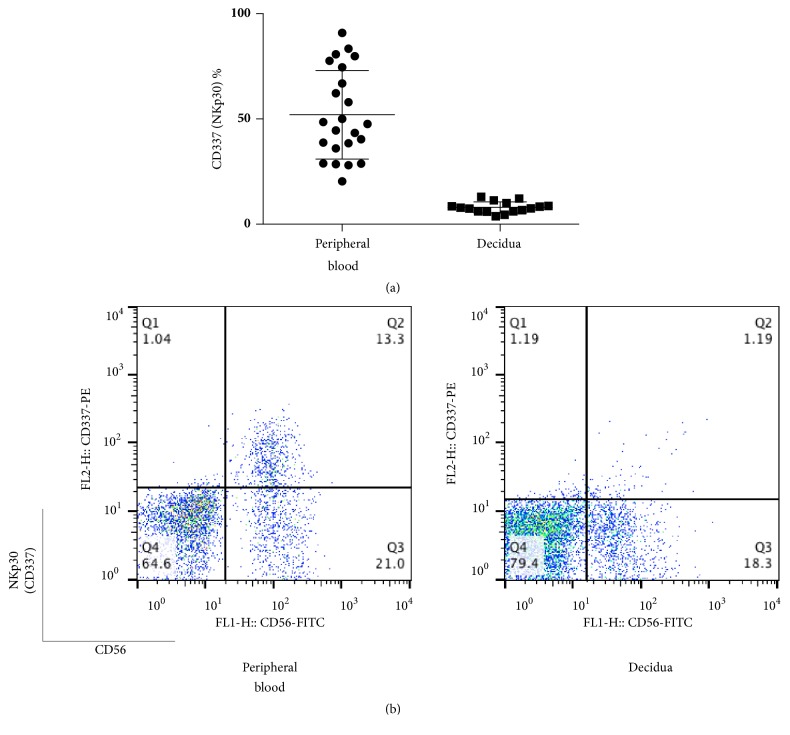
(a) Percentage of CD337 (NKp30)+ cells among CD56+CD3− cells at term pregnancy in peripheral blood and decidua. The percentage of CD337 (NKp30)+ cells in CD56+3- lymphocytes was lower than in peripheral blood. (b) Representative dot plot analysis of CD337 (NKp30)+ cells among CD56+CD3- lymphocytes taken from term pregnancy by flow cytometry. The gate was set around the CD3- lymphocytes to analyze the CD337+CD56+ NK cells.

**Figure 6:**
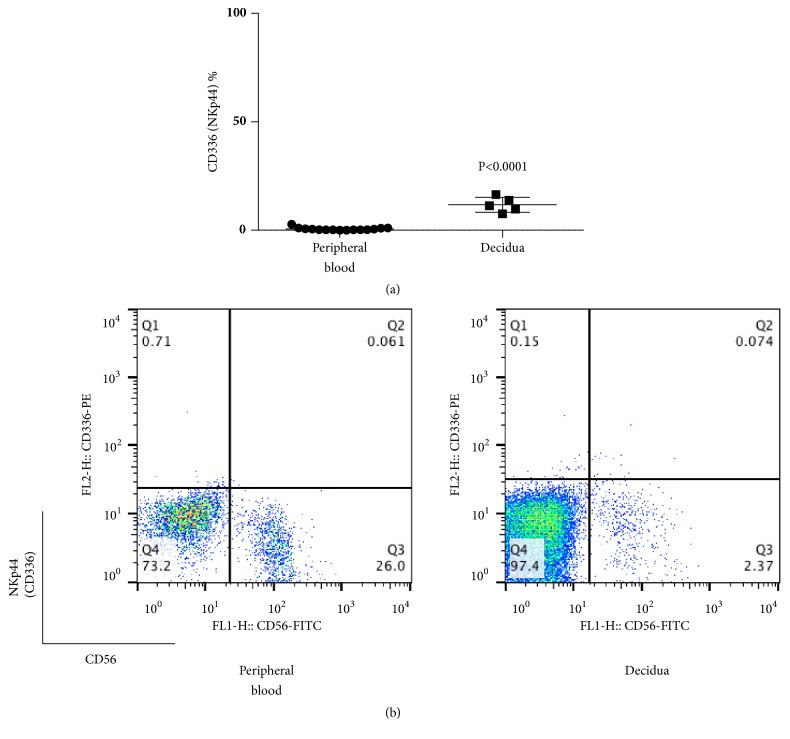
(a) Percentage of CD336 (NKp44)+ cells among CD56+CD3- cells at term pregnancy in peripheral blood and decidua. The percentage of CD336 (NKp44)+ cells among CD56+CD3- lymphocytes was higher than in peripheral blood. (b) Representative dot plot analysis of CD336 (NKp44)+ cells among CD56+CD3- lymphocytes taken from term pregnancy by flow cytometry. The gate was set around the CD3- lymphocytes to analyze the CD336+CD56+ NK cells.

The percentages of NKp30 (CD337)+ cells among CD56+CD3- lymphocyte were 8.0±2.6 and 52.0±21.0 in decidua and peripheral blood, respectively, and the former had values that were lower than the latter (p <0.0001) (Figure 5). Cells that were positive for NKG2D (CD314), NKp46 (CD335), and NKp30 (CD337) among CD56+CD3- cells in the decidua were also lower than those in peripheral blood. The percentages of NKp44 (CD336)+ cells among CD56+CD3- lymphocytes were 11.8±3.5 and 0.6±0.7 in decidua and peripheral blood, respectively (Figure 6), showing higher values for decidua than peripheral blood (p≦0.0001).

### 3.1. Cytokine Production of Peripheral Blood and Decidua Cells in Normotensive Pregnancy

**Figure 7:**
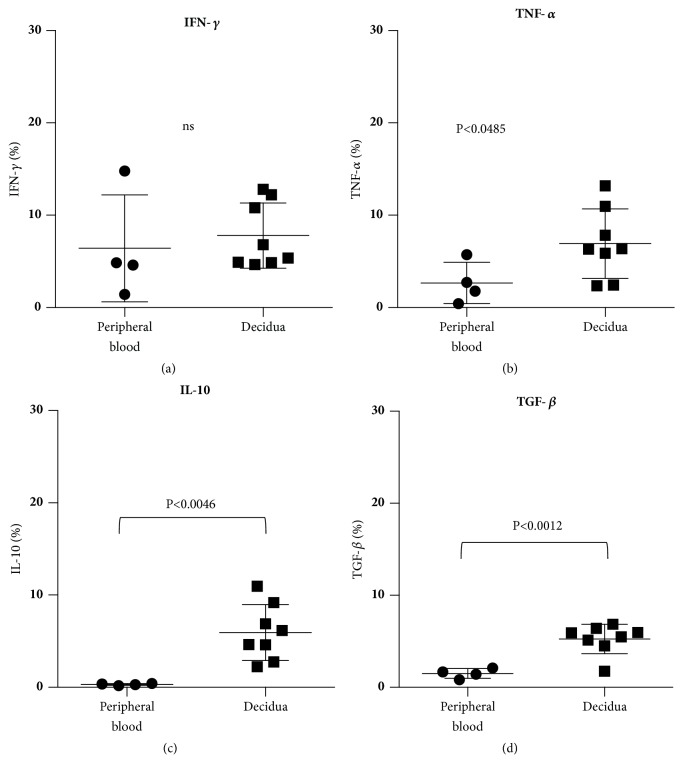
Production of cytokine from CD56+ cells during normal term pregnancy. (a) No significant difference was detected in the percentage of IFN-*γ* producing CD56+ lymphocytes between peripheral blood and decidua. (b) The percentages of TNF-*α* producing CD56+ lymphocytes in decidua were higher than those in peripheral blood. (c) The percentages of IL-10 producing CD56+ lymphocytes in decidua were higher than those in peripheral blood. (d) The percentages of TGF-*β* producing CD56+ lymphocytes in decidua were higher than those in peripheral blood.

The percentages of CD56+ cells from peripheral blood (n=4) and decidua (n=8) that produced IFN-*γ* in normotensive pregnancy, which were 6.4±5.8% and 7.8±3.5%, respectively, showed no significant difference (Figures 7(a)). The percentages of TNF-*α*-producing cells among CD56+ cells from peripheral blood and decidua cells in normotensive pregnancy were 2.7±2.2% and 6.9±3.8%, respectively, with the latter cells having a higher percentage than the former (P<0.0485) (Figure 7(b)). The percentages of IL-10-producing CD56+ cells from peripheral blood and decidua were 0.3±0.1% and 5.9±3.0%, respectively, clearly indicating that the percentage of decidual cells that produce IL-10 was higher than that in peripheral blood (p<0.0046) (Figure 7(c)). The percentages of TGF-*β*-producing CD56+ cells from peripheral blood and decidua were 1.5±0.5% and 5.3±1.6%, respectively, with the decidual cells showing a higher rate than those from peripheral blood (p<0.0012)(Figure 7(d)).

## 4. Discussion

We evaluated the NCRs in decidual CD53+CD3- NK cells during term pregnancy and demonstrated that NCRs of CD56+CD3- NK cells, such as NKp46, NKp30, and NKG2D, are downregulated in the decidua to a greater degree than in peripheral blood. It has been reported that the cytotoxic activity of decidual NK cell is depressed during pregnancy [2–5] and that the suppression of cytotoxicity may be multifactorial and may include both cellular and humoral elements [1, 2]. From our experiment, NK cell cytotoxity may be suppressed because the expression of NCRs is lower in the decidua than in peripheral blood.

Marlin R reported the NCR manifestations of decidual CD56+CD3- NK cells, such as NKp46, NKp30, NKp44, and NKG2D, during the first trimester of pregnancy [9]. In term decidua, the expression of NKp46 and NKp30 in NK cells is lower than in the first trimester, and the expression of NKG2D is higher in the first trimester.

NK cell subtypes during pregnancy have been reported, such as the CD56+CD3-CD16- NK cells and CD56bright CD16- NK cells, known as uterine NK cells, which make up 70-80% of NK cells in the decidua [18]. In our experiment, there were significant increases in CD56+CD3- and CD56+CD3-CD16- NK cells as well as a significant decrease in CD56+CD3-CD16+ NK cells in the decidua compared with peripheral blood in term pregnancy. For the decreased percentages of CD56+CD3-CD16+ NK cells, we reported such decreased percentages of CD56+CD3-CD16+ NK cells in the first-trimester pregnancy [19]. The same pattern of NK cell subtypes is said to exist. However, the percentage of CD56+CD16-NK cells in the decidua at term is lower than that of the first-trimester decidua. This may affect the expression of NCRs. However, the expression of NCRs may vary from the first-trimester decidua to term; further evaluation will be required.

NK receptor ligands are expressed in invading placental trophoblast cells, as reported by Vacca [22]. Moreover, Hanna reported that the ligands of NKp30 and NKp44 are detected within the tissue of the decidua [20]. The decrease in NK receptor expression could be due to increased interactions with their respective ligands, based on a report that ligand binding can induce the downregulation of NK receptors [23, 24]. The downregulation of the NKp30 receptor may thus depend on ligand binding. As the placental weight gain may lead to the increased expression of ligands, the NKp30 receptor may be further downregulated.

Impaired NK cell function in tumors is associated with the downmodulation of NCRs, which is said to be caused by ligand-induced receptor modulation, such as the shedding of ligands from the tumor cell surface, as shown in NKG2D [25]. We can therefore say that the shedding of trophoblast debris from the placenta could lead to the downregulation of the NKG2D receptor [26]. NK cell cytotoxity for fetal tissues may be suppressed because the expression of NCRs decreased more compared with peripheral blood. This may suppress NK cell activity in the decidua at term pregnancy.

It has been reported that the role of cytokines, such as IFN-*γ*, TNF-*α*, IL4, IL10, and TGF-*β*, in the decidua is important for maintaining pregnancy [10–13].

NKp44 is upregulated more in the decidua than in peripheral blood. We think that decidual NKp44 NK cells are activated because NKp44 is expressed only in activated circulating NK cells [7]. We analyzed the cytokine expression in peripheral and decidua CD56+ NK cells. The expression levels of cytokines such as TNF-*α*, IL10, and TGF-*β* increased more in decidual CD56+ cells than in peripheral blood.

The cytokine production of CD56+CD3-CD16- NK cells is higher than that of CD56+CD3-CD16+ NK cells, thus possibly giving the former a role in the maintenance of pregnancy [16]. Due to the increased expression of NKp44, cytokine expression may increase more compared with peripheral blood in CD56+ cells. The cytokine expressions that originated from NKp44 may play a role in maintaining pregnancy at term. We need to analyze the cytokine expression of NCR-positive cells in CD56+CD3-CD16+ and CD56+CD3-CD16- NK cells.

In term normal pregnancy, the expression of some NCRs in the decidua decreased more than that in peripheral blood, which may be related to the decreased NK cell cytotoxicity. However, as some functions of NK cells are activated and consequently produce some cytokines, an appropriate balance of NK cell cytokine production and NK cell cytotoxicity may be produced and may play a role in maintaining pregnancy at term.

## References

[1] Warning J. C., McCracken S. A., Morris J. M. (2011). A balancing act: Mechanisms by which the fetus avoids rejection by the maternal immune system. *Reproduction*.

[2] Gregory C. D., Shah L. P., Lee H., Scott I. V., Golding P. R. (1985). Cytotoxic reactivity of human natural killer (NK) cells during normal pregnancy: a longitudinal study. *Journal of Clinical & Laboratory Immunology*.

[3] Chao K., Yang Y., Ho G. (1995). Decidual Natural Killer Cytotoxicity Decreased in Normal Pregnancy but Not in Anembryonic Pregnancy and Recurrent Spontaneous Abortion. *American Journal of Reproductive Immunology*.

[4] Manaseki S., Searle R. F. (1989). Natural killer (NK) cell activity of first trimester human decidua. *Cellular Immunology*.

[5] King A., Birkby C., Loke Y. W. (1989). Early human decidual cells exhibit NK activity against the K562 cell line but not against first trimester trophoblast. *Cellular Immunology*.

[6] Ponte M., Cantoni C., Biassoni R. (1999). Inhibitory receptors sensing HLA-G1 molecules in pregnancy: Decidua-associated natural killer cells express LIR-1 and CD94yNKG2A and acquire p49, an HLA-G1-specific receptor. *Immunology*.

[7] Kruse P. H., Matta J., Ugolini S., Vivier E. (2014). Natural cytotoxicity receptors and their ligands. *Immunology & Cell Biology*.

[8] Horton N. C., Mathew P. A. (2015). NKp44 and Natural Cytotoxicity Receptors as Damage-Associated Molecular Pattern Recognition Receptors. *Frontiers in Immunology*.

[9] Marlin R., Duriez M., Berkane N. (2012). Dynamic Shift from CD85j/ILT-2 to NKG2D NK Receptor Expression Pattern on Human Decidual NK during the First Trimester of Pregnancy. *PLoS ONE*.

[10] Bulmer J. N., Lash G. E. (2005). Human uterine natural killer cells: a reappraisal. *Molecular Immunology*.

[11] Saito S., Nishikawa K., Morii T. (1993). Cytokine production by CD16. *International Immunology*.

[12] Sharma S., Godbole G., Modi D. (2016). Decidual Control of Trophoblast Invasion. *American Journal of Reproductive Immunology*.

[13] Clark D. A., Vince G., Flanders K. C., Hirte H., Starkey P. (1994). Immunology: CD56+ lymphoid cells in human first trimester pregnancy decidua as a source of novel transforming growth factor-*β*2-related immunosuppressive factors. *Human Reproduction*.

[14] Fukui A., Funamizu A., Yokota M. (2011). Uterine and circulating natural killer cells and their roles in women with recurrent pregnancy loss, implantation failure and preeclampsia. *Journal of Reproductive Immunology*.

[15] Yokota M., Fukui A., Funamizu A. (2013). Role of NKp46 Expression in Cytokine Production by CD56-Positive NK Cells in the Peripheral Blood and the Uterine Endometrium. *American Journal of Reproductive Immunology*.

[16] Campbell K. S., Hasegawa J. (2013). Natural killer cell biology: an update and future directions. *The Journal of Allergy and Clinical Immunology*.

[17] Starkey P. M., Sargent I. L., Redman C. W. (1988). Cell populations in human early pregnancy decidua: characterization and isolation of large granular lymphocytes by flow cytometry. *Immunology*.

[18] Nishikawa K., Saito S., Morii T. (1991). Accumulation of CD16. *International Immunology*.

[19] Yamamoto T., Takahashi Y., Kase N., Mori H. (1999). Role of decidual natural killer (NK) cells in patients with missed abortion: differences between cases with normal and abnormal chromosome. *Clinical & Experimental Immunology*.

[20] Hanna J., Goldman-Wohl D., Hamani Y. (2006). Decidual NK cells regulate key developmental processes at the human fetal-maternal interface. *Nature Medicine*.

[21] Petrović O., Gudelj L., Rubeša G., Haller H., Beer A. E., Rukavina D. (1994). Decidual-trophoblast interactions: decidual lymphoid cell function in normal, anembryonic, missed abortion and ectopic human pregnancy. *Journal of Reproductive Immunology*.

[22] Vacca P., Cantoni C., Prato C. (2008). Regulatory role of NKp44, NKp46, DNAM-1 and NKG2D receptors in the interaction between NK cells and trophoblast cells. Evidence for divergent functional profiles of decidual versus peripheral NK cells. *International Immunology*.

[23] Groh V., Wu J., Yee C., Spies T. (2002). Tumour-derived soluble MIC ligands impair expression of NKG2D and T-cell activation. *Nature*.

[24] Sandusky M., Messmer B., Watzl C. (2006). Regulation of 2B4 (CD244)-mediated NK cell activation by ligand-induced receptor modulation. *European Journal of Immunology*.

[25] Moretta L., Montaldo E., Vacca P. (2014). Human Natural Killer Cells: Origin, Receptors, Function, and Clinical Applications. *International Archives of Allergy and Immunology*.

[26] Covone A. E., Johnson P. M., Mutton D., Adinolfi M. (1984). Trophoblast cells in peripheral blood from pregnant women. *The Lancet*.

